# Implementation of non-pharmacological recommendations and its associated factors among hypertensive patients on follow-up in hospitals of South Omo and Gofa zones, Southern Ethiopia: A facility based cross sectional study

**DOI:** 10.1371/journal.pgph.0006604

**Published:** 2026-06-10

**Authors:** Tesfaye Kanko, Zelalem Kofole, Yohannes Simon, Lankamo Ena, Melkamu Merid

**Affiliations:** 1 Department of Biomedical Sciences, College of Medicine and Health Sciences, Arba Minch University, Arba Minch, Ethiopia; 2 Department of Internal Medicine, College of Medicine and Health Sciences, Arba Minch University, Arba Minch, Ethiopia; 3 Department of Nursing, College of Medicine and Health Sciences, Arba Minch University, Arba Minch, Ethiopia; 4 Department of Public Health, College of Medicine and Health Sciences, Arba Minch University, Arba Minch, Ethiopia; Sun Yat-Sen University, CHINA

## Abstract

Hypertension is a major public health issue and inadequately controlled in a large proportion of patients globally. It can be adequately managed both pharmacologically and non-pharmacologically. This study aimed to assess the implementation of non-pharmacological recommendations and its associated factors among hypertensive patients attending hospitals in the South Omo and Gofa zones, southern Ethiopia. A health facility-based cross-sectional study was conducted among hypertensive patients on follow-up at the chronic care unit of Jinka and Sawla general hospitals in the South Omo zone (currently the Ari zone) and the Gofa zone, southern Ethiopia, from June 01 to July 30, 2025. The participants were selected using proportionate allocation and a simple random sampling method. A pretested and interviewer-administered structured questionnaire adapted from a validated tool to measure hypertension self-care and the Joint National Committee recommendations on the treatment of hypertension was utilized to collect the data using Kobo Toolbox. Statistical Package for Social Sciences (SPSS) version 25 was utilized for data analysis. Descriptive statistics such as frequency and cross-tabulation were used to summarize the data. Binary logistic regression was computed to identify determinants of hypertensive patients’ implementation of non-pharmacological recommendations. Significance was declared at p-value<0.05. In this study, majority of the participants were male and aged above 50 years. This study revealed that 58.9% of participants had good overall implementation of non-pharmacological recommendations for hypertension management. In this study participant age (AOR: 3.64; 95% CI: 1.43-9.28), educational level (AOR: 3.84; 95% CI: 1.42-10.35), monthly income (AOR: 0.57; 95% CI: 0.34-0.96) and knowledge level about non-pharmacological recommendations (AOR: 5.02; 95% CI: 2.94-8.57) were found to be significant determinants of hypertensive patients’ implementation of non-pharmacological recommendations (p < 0.05). This study identified a considerable magnitude of poor implementation of non-pharmacological recommendations for hypertension management among hypertensive patients. The participant’s age, educational level, monthly income, and knowledge level were found to be significant predictors of the implementation of recommendations among patients. Therefore, establishing a facility-based patient peer support club, considering digital health (automated SMS reminder) intervention, awareness creation campaigns and patient centered health education would play an indispensable role in improving the implementation of recommendations among hypertensive patients.

## Introduction

Hypertension is a common chronic non-communicable disease and a major cause of premature death worldwide. Currently, it is a global challenge in both developed and developing countries. Globally, an estimated 1.4 billion (33%) of adults had hypertension, and over three-fourths of adults with hypertension live in low and middle income countries [1]. A systematic meta-analysis in Ethiopia showed about 20.63% prevalence of hypertension, with a higher proportion in urban than rural areas [2].

Hypertension contributes to the burden of heart disease, stroke, and kidney failure, hence to premature mortality and disability [3]. According to the World Health Organization (WHO), globally cardiovascular disease accounts for approximately 17 million deaths a year. Of these, hypertension-related complications account for 9.4 million deaths worldwide every year [3]. Increments in the prevalence of hypertension and other cardiovascular diseases in the developing countries have been associated with aging of populations, urbanization, and socioeconomic changes favoring sedentary habits [3].

Hypertension can be adequately managed pharmacologically, and its deleterious contributing factors can be successfully countered by implementing non-pharmacological recommendations [4–8].

Effective hypertension management requires weight loss for obese patients, sodium reduction not exceeding one and a half grams daily, alcohol intake reduction, smoking cessation, avoidance of excessive caffeine, and diet modification such as low intake of saturated and total fat and increased intake of fruits and vegetables [9,10]. Hypertensive patients, irrespective of their disease stage, are encouraged to adopt these non-pharmacological measures, which supplement pharmacological hypertension management [11].

Despite the availability of safe and effective antihypertensive medications and the existence of clear treatment guidelines, hypertension is still inadequately controlled in a large proportion of patients worldwide [12].

Literatures indicates that about half of aware hypertensive cases do not take treatment, and more than half of those treated have uncontrolled blood pressure [13,14]. Several factors relating to patients, physicians, and the health care system are responsible for poor treatment and suboptimal control of hypertension [15,16]. Unawareness of non-pharmacological therapies and failure to implement these were among the identified patient-related barriers to blood pressure control [17].

To our knowledge, there is no previous study reporting hypertensive patients’ implementation of this important strategy and its associated factors in the current study area. Therefore, this study aimed to assess the implementation of non-pharmacological recommendations and its determinant factors among hypertensive patients on follow-up in hospitals of South Omo and Gofa zones. The findings of this study would have paramount importance to the effective control of elevated blood pressure and prevention of its sequelae in hypertensive patients.

## Methods and population

### Study design, population, setting and period

A health-facility based cross-sectional study was conducted among adult hypertensive patients on follow-up in the chronic care units at Jinka and Sawla General Hospitals located in the South Omo (currently Ari) zone and Gofa zone in southern Ethiopia from June 01 to July 30, 2025. Jinka General Hospital (located in South Omo Zone, Jinka town at 750 km far from Addis Ababa) and Sawla General Hospital (located in Gofa Zone, Sawla town at 464 km far from Addis Ababa) are the two high-volume general hospitals providing comprehensive services to the local residents and managing referrals from area clinics, health centers, and primary hospitals.

### Inclusion and exclusion criteria

All adult hypertensive patients on follow up were included, whereas newly diagnosed patients and those critically ill and unable to communicate were excluded from this study.

### Sample size determination

The sample size was determined by Open Epi version 3, open source, using single population proportion formula. To calculate the sample size, we utilized a proportion of 33.3% from previous study done in southwest Ethiopia [18], a 95% confidence level (Z α/2 = 1.96), and a 5% margin of error. This calculation resulted in a sample size of 342. Accounting for a 10% non-response rate, the final estimated sample size was adjusted to 376.

### Sampling procedure

The sampling procedure involved proportional allocation based on the respective total number of registered hypertensive patients on follow-up at Jinka (570 patients) and Sawla (450 patients) general hospitals. This resulted in an allocated sample of 210 from Jinka and 166 from Sawla general hospital. Finally, participants were selected using a simple random sampling method from the sampling frame, which was prepared by taking the list of registered hypertensive patients on follow-up at each hospital.

### Study variables

**Dependent variable**

- Implementation of non-pharmacological recommendations: Good/Poor

**Independent variables**

- Sociodemographic characteristics (age, sex, marital status, monthly income, education level, occupation, residence, family support)
- Information access/source of information about non-pharmacological management (Health professional, social media, broadcast (livestreaming media)
- Medical factors (Duration of diagnosis, family history of hypertension, presence of co-morbidity, regular visit, health education)
- Personal factors (Knowledge, attitude on hypertension non-pharmacological management)

### Data collection tool and procedure

An interviewer administered a structured questionnaire adapted from related studies, and a validated tool [6,8,19–21] was used for data collection. The non-pharmacological strategy implementation was assessed using questionnaires adapted from validated tools to measure hypertension self-care [21], non-pharmacological domains for hypertension management recommended by the Joint National Committee on Detection, Prevention, Evaluation and Treatment of Hypertension (JNC7) [8], and the WHO Stepwise Approach to Surveillance (STEPS) of Non-Communicable Diseases [20].

The questionnaire consisted of five sections designed to assess the participants’ socio-demographics, medical history, information sources, knowledge, and attitude concerning non-pharmacological hypertension management, along with their implementation of the recommended strategies. Participants’ height was measured barefoot using a portable stadiometer and recorded to the nearest point five centimeters. Weight was determined using a digital scale and recorded to the nearest point one kilogram. Data were collected at each hospital by two trained BSc. nurses using the digital platform Kobo Toolbox, with the entire operation being overseen and supervised by the investigators.

### Data management and quality control

To ensure the data quality, the questionnaire was first prepared in English and translated into Amharic, which is a widely spoken language in the study area, and then back-translated into English to ensure its consistency by bilingual language experts. A pretest was conducted in Arba Minch General Hospital by taking 5% of the sample size to check for the logical order of the questions and the presence of vague words and unclear issues. The questionnaire was modified and refined based on feedback from the pretest before being used for the main data collection. Two days of training were given for the data collectors on the overall aim of the study, the contents of the questionnaire and questions, and how to approach and interview the respondents. In addition, practical training was given on how to access the questionnaire and collect, save, and send the data to the server using Kobo Toolbox. Investigators performed daily checks on the collected data to ensure completeness, taking prompt action to address any issues.

### Data processing and statistical analysis

The collected data were downloaded from the Kobo Toolbox server and checked for completeness, cleaned thoroughly, coded appropriately using a Microsoft Excel spreadsheet, and then exported to SPSS version 25 for statistical analysis.

Descriptive statistics such as cross-tabulation and frequency were used to summarize the data. Binary logistic regression analysis was performed to identify factors associated with the implementation of non-pharmacological recommendations. Multicollinearity among independent variables was ruled out, as indicated by acceptable Variance Inflation Factor (VIF) values below 5. The model fitness was confirmed by the Hosmer and Lemeshow test with a p-value > 0.05. Those variables with a p-value < 0.3 in the bivariable logistic regression model were considered as candidates for the multivariable logistic regression model, and a corresponding p-value < 0.05 with an adjusted odds ratio at a 95% confidence interval (CI) was considered to declare the presence of a significant association between the outcome and independent variables. The study findings were presented using narratives, tables, and graphs, depending on the type of data.

### Operational definitions

Regarding the level of implementation of non-pharmacological recommendations (Dietary sodium reduction, Adopt DASH eating plan, physical activity, moderation/abstaining of alcohol consumption, cessation of smoking and weight reduction): Nine items were used to assess the patient’s implementation of the recommendations and each correct response is coded as 1 and incorrect as 0 for computing the mean score. Those respondents who correctly answered above the mean score, which was 5.8 were considered as having good overall implementation, whereas those who answered below the mean value were considered as having poor overall implementation.

Dietary sodium reduction: Avoiding salty food or consuming less than six grams or one teaspoon of table salt per day [22]. Adopt DASH eating plan: Consume a diet rich in fruits, vegetables, and low-fat dairy products with a reduced content of saturated and total fat [22]. Physical activity: Engage in regular aerobic physical activity such as brisk walking, jogging for at least 30 min per day, most days of the week [22].

Abstaining/moderating alcohol drinking: those who do not drink alcoholic beverages in the last seven days of the week or not at all are taken as abstainers. Cessation of smoking: those who do not smoke cigarettes in the last seven days of the week or not at all were taken as nonsmokers.

Weight reduction: Maintain normal body weight (body mass index 18.5–24.9 kg/m2) [22].

Regarding knowledge to non-pharmacological management of hypertension: Eight items with a response of yes or no were used to assess the knowledge about recommendations. Participants who correctly answered above the mean score, which was 6.9, were considered as having good knowledge, whereas those who answered below the mean score were considered as having poor knowledge. Regarding attitude towards hypertension non-pharmacological management: Attitude assessed by six items containing a Likert scale with a total response value of 6–30. Respondents with a response score of 24 and above were considered as having a positive attitude, whereas those who scored below 24 were considered as having a negative attitude.

### Ethical approval and consent to participate

This study was conducted in accordance with the Declaration of Helsinki statement of ethical principles for medical research involving human participants. Before commencing data collection, ethical approval was obtained from the Arba Minch University College of Medicine and Health Science Institutional Research Ethics and Review Board with reference number IRB/1377/2023. In addition, all study participants were informed about the study’s objectives and procedures, and written informed consent was obtained from each of the study participant before the actual data collection.

## Results

### Sociodemographic characteristics of the study participants

The data were collected from 365 study participants with a response rate of 97.1%. Among the study participants, the majority were male, urban dwellers, aged above 50 years and married. Regarding religion, occupation, educational level and monthly income, the majority of the study participants were Orthodox, government employees, had achieved a college or higher educational level, and had a monthly income of less than 5000 ETB (Table 1).

**Table 1:** Sociodemographic characteristics of the study participants (n = 365).

Variables	Category	Frequency	Percentage (%)
Sex	Male	190	52.1
	Female	175	47.9
Residence	Urban	265	72.6
	Rural	100	27.4
Age	30-39	27	7.4
	40-49	71	19.4
	50-59	136	37.3
	>=60	131	35.9
Educational level	Cannot read and write	83	22.7
	Read and write only	35	9.6
	Primary school	41	11.2
	Secondary school	69	18.9
	College and above	137	37.5
Marital status	Single	14	3.8
	Married	278	76.2
	Divorced	37	10.1
	Widowed	36	9.9
Occupation	Housewife	68	18.6
	Farmer	88	24.1
	Private employee	54	14.8
	Government employee	108	29.6
	Merchant	35	9.6
	Others*	12	3.3
Religion	Orthodox	167	45.7
	Protestant	150	41.1
	Catholic	8	2.2
	Muslim	40	11
Monthly income(ETB)	<=5000	179	49
	5001-10000	107	29.3
	10001-15000	57	15.6
	>=15000	22	6

*Retired, Priest, Student.

### Medical history and source of information about non-pharmacological management of hypertension

The majority of the study participants had less than five years’ duration after being diagnosed with hypertension, and about half of the participants had a family history of hypertension. Sixty seven percent of participants had at least one diagnosed comorbidity, such as heart disease, kidney disease, liver disease, diabetes mellitus and asthma. About 97% of the study participants responded that they took their prescribed antihypertensive drugs regularly, got health education from their healthcare provider, and had information about non-pharmacological recommendations. Most of the study participants had received information from health professionals, visited healthcare providers regularly, and had family support to implement non-pharmacological recommendations (Table 2).

**Table 2:** Study participants medical history and source of information about non-pharmacological management of hypertension (n = 365).

Variables	Category	Frequency	Percentage (%)
Duration after diagnosed hypertension (years)	< 5	233	61.1
	5-10	119	32.6
	>10	23	6.3
Family history of hypertension	No	181	49.6
	Yes	184	50.4
Have diagnosed comorbidity	No	245	67.1
	Yes	120	32.9
Did you take your prescribed drugs regularly?	No	12	3.3
	Yes	353	96.7
Have you heard about non-pharmacological recommendations?	No	11	3
	Yes	354	97
Source of information	Health-professional	305	83.6
	Mass media	15	4.1
	Family member	22	6
	Friends/colleagues	12	3.3
Did you get health education from your healthcare provider?	No	10	2.7
	Yes	355	97.3
Did you visit your healthcare provider regularly?	No	38	10.4
	Yes	327	89.6
Did you get family support to implement non-pharmacological recommendations?	No	52	14.2
	Yes	313	85.8

### Knowledge about non-pharmacological recommendations of hypertension management

**Fig 1:**
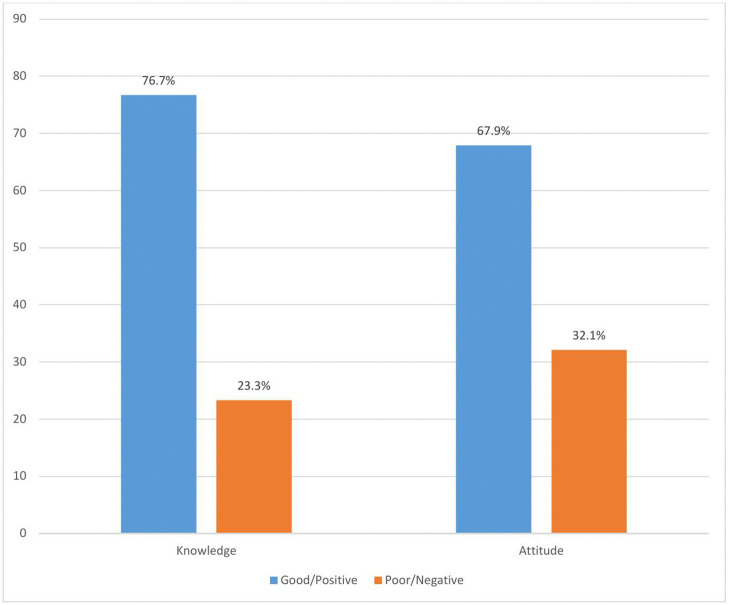
Study participant’s Knowledge and attitude towards non-pharmacological recommendations for hypertension management (n = 365).

Of the total, more than three-quarters of the study participants had good knowledge about the non-pharmacological recommendations of hypertension management (Fig 1).

Most of the study participants recognized that factors, such as high salt and saturated fat intake, being overweight, drinking alcohol, and smoking can increase hypertension risk, while regular physical exercise and a diet rich in fruits, vegetables, fish, and poultry lower hypertension risk (Table 3).

**Table 3:** Study participants’ knowledge about non-pharmacological recommendations for hypertension management (n = 365).

Variables	Category	Frequency	Percentage (%)
Do you think that high salt intake can increase the risk of high blood pressure?	No	2	0.5
	Yes	363	99.5
Do you think that eating food high in saturated fat can increase risk of high blood pressure?	No	42	11.5
	Yes	323	88.5
Do you think that regular intake of fish, poultry, vegetable oils can lower blood pressure?	No	119	32.6
	Yes	246	67.4
Do you think that eating fruits, vegetables can lower blood pressure?	No	8	2.2
	Yes	357	97.8
Do you think that physical exercise for 30 minutes at least four days a week can lower blood pressure?	No	69	18.9
	Yes	296	81.1
Do you think that being overweight can increases the risk of high blood pressure?	No	57	15.6
	Yes	308	84.4
Do you think that alcohol drinking can increase risk of high blood pressure?	No	31	8.5
	Yes	334	91.5
Do you think that cigarette smoking can increase risk of high blood pressure?	No	70	19.2
	Yes	295	80.8

### Attitude towards non-pharmacological recommendations of hypertension management

Over two-thirds of participants expressed a favorable attitude toward non-pharmacological hypertension management (Fig 1). The majority of study subjects strongly agreed that controlling diet, salt intake, and alcohol drinking is important for blood pressure control. The other majority agreed that maintaining normal body weight, doing regular physical exercise, and quitting smoking are important for controlling blood pressure (Table 4).

**Table 4:** Study participants’ attitude towards non-pharmacological recommendations for hypertension management (n = 365).

Variables	Category N (%)				
	Strongly disagree	Disagree	Neutral	Agree	Strongly agree
Controlling diet is important for blood pressure control.	2 (0.5)	–	35 (9.6)	171 (46.8)	157 (43)
Controlling salt intake is important for blood pressure control	1 (0.3)	–	5 (1.4)	63 (17.3)	296 (81.1)
Maintaining normal body weight is important for controlling blood pressure	7 (1.9)	42 (11.5)	106 (29)	159 (43.6)	51 (14)
Doing regular physical exercise is important for controlling blood pressure	5 (1.4)	10 (2.7)	137 (37.5)	153 (41.9)	60 (16.4)
Reducing alcohol intake is important for controlling blood pressure	1 (0.3)	1 (0.3)	58 (15.9)	102 (27.9)	203 (55.6)
Avoiding cigarette smoking is important for controlling blood pressure	1 (0.3)	25 (6.8)	115 (31.5)	152 (41.6)	72 (19.7)

### Implementation of non-pharmacological recommendations of hypertension management

**Fig 2:**
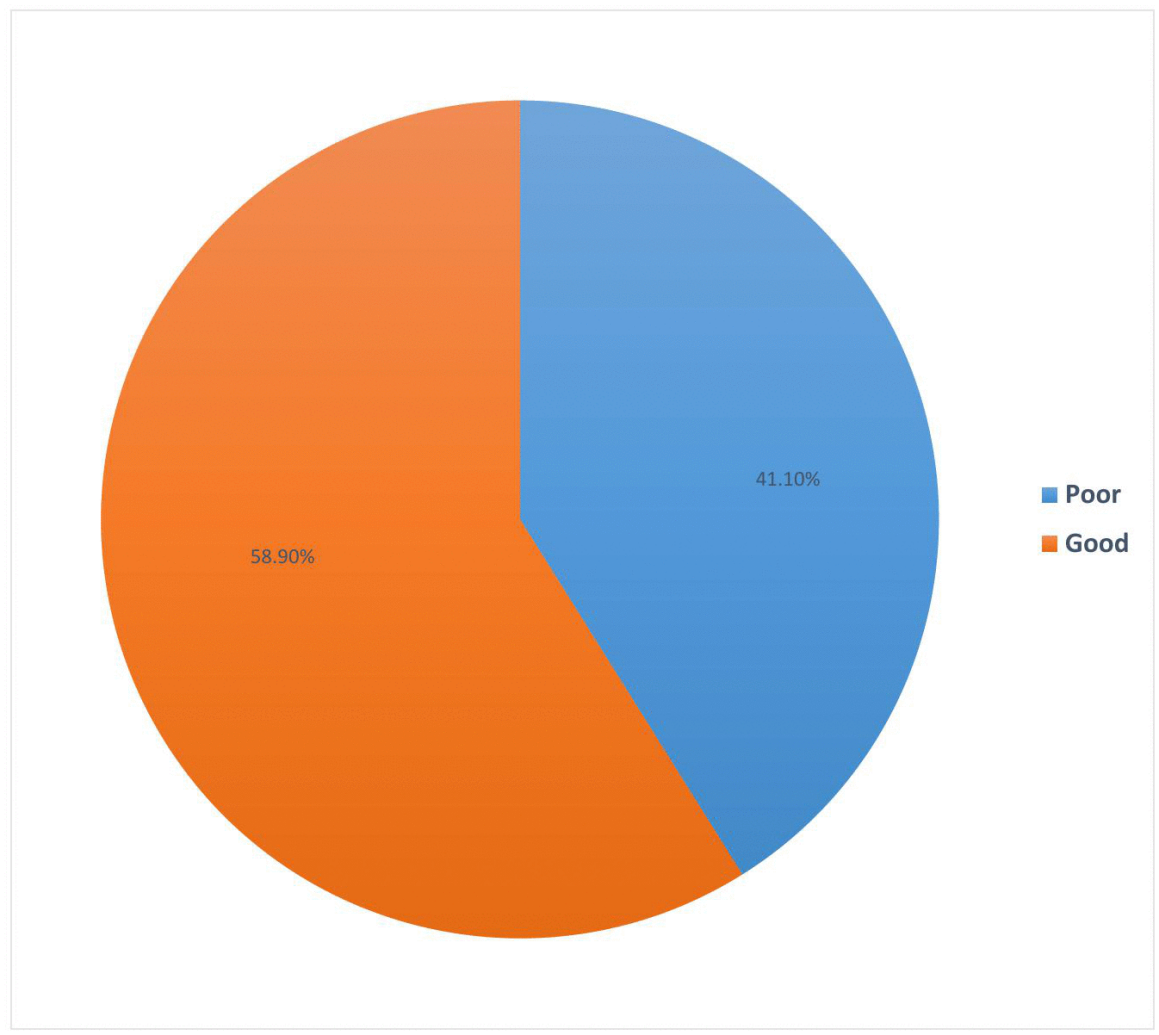
Study participant’s implementation of non-pharmacological recommendations for hypertension management (n = 365).

Of the total, 215 (58.9) participants had a good overall implementation of non-pharmacological recommendations of hypertension management (Fig 2).

The majority of the study participants maintained strict dietary habits, which included never adding salt to prepared meals and avoiding foods high in either salt or saturated fats/butter. Of the participants, 129 (35.3) performed specific physical exercise (such as walking, jogging, playing football, swimming, or biking), and 99 (27%) did the activity for at least 30 minutes per day for at least three days a week. The majority of the respondents abstained from drinking alcoholic beverages and smoking cigarettes and maintained their normal body weight (Table 5).

**Table 5:** Study participants’ implementation of non-pharmacological recommendations for hypertension management (n = 365).

Variables	Category	Frequency	Percentage
How often do you add salt to your food before eating or while eating?	Always	1	0.3
	Usually	14	3.8
	Sometimes	129	35.3
	Never	221	60.5
How often salt is added when cooking foods in your household?	Always	25	6.8
	Usually	24	6.6
	Sometimes	230	63
	Never	86	23.6
How often do you eat salty food?	Always	1	0.3
	Usually	11	3
	Sometimes	118	32.3
	Never	235	64.4
How often do you consume foods that contain saturated fat/butter?	Always	1	0.3
	Usually	1	0.3
	Sometimes	115	31.5
	Never	248	67.9
How often do you include fruits, vegetables in your diet?	Always	50	13.7
	Usually	212	58.1
	Sometimes	103	28.2
	Never	–	–
Do you perform specific physical exercise other than what you do as part of your work?	No	236	64.7
	Yes	129	35.3
How many days in the past one week did you do physical exercise for at least 30 minutes?	1-2	30	8.22
	3	31	8.5
	>=4	68	18.63
Did you ever or in the last 7 days drink any alcoholic beverage?	No	270	74
	Yes	95	26
Did you ever or in the last 7 days smoke cigarette?	No	344	94.2
	Yes	21	5.8
Maintain body weight(BMI)(Kg/m2)	Underweight	13	3.6
	Normal weight	221	60.5
	Overweight	120	32.9
	Obese	11	3

### Factors associated with implementation of non-pharmacological recommendations for hypertension management

In a bivariable logistic regression, crude associations were observed for educational level, information access, family support, regular healthcare visits, and knowledge regarding non-pharmacological recommendations.

A multivariable logistic regression model was computed, using variables with p < 0.3 in the bivariable analysis to control for confounders. Finally, participants’ age, educational level, monthly income, and knowledge of non-pharmacological recommendations were found to be statistically significant predictors of the outcome variable (p < 0.05). Participants with higher monthly income had lower odds of good implementation of the recommendations (Table 6).

**Table 6:** Factors associated with implementation of non-pharmacological recommendations for hypertension management among hypertensive patients on follow up (n = 365).

Variables	Category	Implementation		COR(95%CI)	P-value	AOR(95%CI)	P-value
		Poor N (%)	Good N (%)				
Sex	Male	75 (20.5)	115 (31.5)	1.15 (0.76- 1.75)	0.51		
	Female	75(20.5)	100(27.4)	1			
Residence	Urban	109(29.9)	156(42.7)	0.99(0.62-1.59)	0.98		
	Rural	41(11.2)	59(16.2)	1			
Age	30-39	18(4.9)	9(2.5)	1	**0.01**	1	0.04*
	40-49	31(8.5)	40(10.9)	2.58(1.02-6.52)		2.59(0.97-6.94	
	50-59	43(11.8)	93(25.5)	4.33(1.79-10.4)		**3.64(1.43-9.28)**	
	>=60	58(15.9)	73(20)	2.52(1.05-6.02)		**2.66(1.05-6.72)**	
Educational level	Cannot read and write	44(12.1)	39(10.7)	1	**0.01**	1	0.04*
	Read and write only	16(4.4)	19(5.2)	1.34(0.61-2.96)		1.43(0.59-3.42)	
	Primary school	8(2.2)	33(9)	4.65(1.92-11.27)		**3.84(1.42-10.35)**	
	Secondary school	24(6.6)	45(12.3)	2.11(1.09-4.08)		1.87((0.86-4.05)	
	College and above	58(15.9)	79(21.6)	1.54(0.89-2.66)		**2.38(1.06-5.36)**	
Marital status	Single	7(1.9)	7(1.9)	1	0.48		
	Married	109(29.9)	169(46.3)	1.55(0.53-4.54)			
	Divorced	19(5.2)	18(4.9)	0.95(0.28-3.24)			
	Widowed	15(4.1)	21(5.8)	1.40(0.41-4.83)			
Occupation	Housewife	24(6.6)	44(12.1)	1	0.55		
	Farmer	39(10.7)	49(13.4)	0.68(0.38-1.32)			
	Private employee	19(5.2)	35(9.6)	1.00(0.48-2.12)			
	Government employee	46(12.6)	62(16.9)	0.74(0.39-1.38)			
	Merchant	18(4.9)	17(4.7)	0.52(0.23-1.18)			
	Other	4(1.1)	8(2.2)	1.09(0.29-4.00)			
Monthly income	<=5000	62(17.0)	117(32.0)	1	**0.10**	1	0.03*
	5001-10000	49(13.4)	58(15.9)	0.63(0.38-1.02)		**0.57(0.34-0.96)**	
	10001-15000	28(7.7)	29(8.0)	0.55(0.30-1.29)		**0.49(0.26-0.94)**	
	>15000	11(3.0)	11(3.0)	0.53(0.22-1.29)		0.44(0.17-1.12)	
Duration after diagnosed hypertension(year)	<5	84(23.0)	139(38.1)	1	**0.21**	1	0.86
	5-10	54(14.8)	65(17.8)	0.73(0.46-1.14		0.86(0.50-1.48)	
	>10	12(3.3)	11(3.0)	0.55(0.23-1.31)		0.96(0.35-2.67)	
Family history of hypertension	No	74(20.3)	107(29.3)	1	0.94		
	Yes	76(20.8)	108(29.6)	0.98(0.65-1.49)			
Diagnosed comorbidity	No	96(26.3)	149(40.8)	1	**0.28**	1	0.08
	Yes	54(14.8)	66(18.1)	0.79(0.51-1.23)		0.64(0.39-1.06)	
Take prescribed drugs regularly	No	6(1.6)	6(1.6)	1	0.52		
	Yes	144(39.5)	209(57.3)	1.45(0.46-4.59)			
Heard information about non-pharmacological recommendations	No	8(2.2)	3(0.8)	1	**0.04**	1	0.08
	Yes	142(38.9)	212(58.1)	3.98(1.04-15.26)		3.55(0.84-15.08)	
Source of information about recommendations	Health professionals	111(30.4)	194(53.2)	2.45(0.76-7.89)	0.64		
	Mass media	13(3.6)	2(0.5)	0.22(0.03-1.41)			
	Family member	11(3.0)	11(3.0)	1.40(0.34-5.79)			
	Friend/colleagues	7(1.9)	5(1.4)	1			
Get health education from health care provider	No	6(1.6)	4(1.1)	1	**0.23**	1	0.38
	Yes	144(39.5)	211(57.8)	2.19(0.61-7.93)		1.87(0.45-7.81)	
Get family support	No	32(8.8)	20(5.5)	1	**0.00**	1	0.16
	Yes	118(32.3)	195(53.4)	2.64(1.45-4.84)		1.72(0.81-3.68)	
Visit your healthcare provider regularly	No	24(6.6)	14(3.8)	1	**0.00**	1	0.12
	Yes	126(34.5)	201(55.1)	2.74(1.36-5.48)		1.88(0.86-4.14)	
Knowledge about recommendations	Poor	59(16.2)	26(7.1)	1	**0.00**	1	0.00*
	Good	91(24.9)	189(51.8)	4.71(2.79-7.97)		5.02(2.94-8.57)	
Attitude towards recommendations	Negative	53(14.5)	64(17.5)	1	**0.26**	1	0.18
	Positive	97(26.6)	151(41.4)	1.29(0.83-2.01)		0.68(0.38-1.20)	

*Statistically significant; CI: Confidence Interval; COR: Crude Odds Ration; AOR: Adjusted Odds Ratio.

## Discussion

This study was intended to assess the implementation of non-pharmacological recommendations and its determinant factors among hypertensive patients attending hospitals in southern Ethiopia. Effective implementation of non-pharmacological recommendations can counteract risk factors contributing to hypertension [4–7]. In the current study, most of the hypertensive patients on follow-up had good overall implementation of the non-pharmacological recommendations for hypertension management. The observed finding is comparable with the report of studies done in India, Lagos, and northwestern Nigeria, which reported good implementation rates of non-drug hypertension control strategies at 54.7%, 52.6%, and 56.7%, respectively [19,23,24]. This finding is lower than the reports of studies conducted in Nekemte (68.9%) and Bishoftu (61.4%), Ethiopia [25,26].

The current finding is noticeably better than the reports from Iran and various Ethiopian regions, where implementation rates were significantly lower, ranging from 23% to 49.6% [6,18,27–30]. This variation might be due to educational level, economic status, living standard, and cultural differences determining lifestyle among the study participants in the study settings. In addition, the variation could be due to differences in the level of knowledge and awareness about non-pharmacological recommendations and their importance for effective management of hypertension.

In the current study, participants aged 50–59 years had higher odds (AOR: 3.64; 95% CI: 1.43-9.28) of implementing the non-pharmacological recommendations than those aged 30–39 years. Similarly, those aged greater than or equal to sixty years were 2.66 times (AOR: 2.66; 95% CI: 1.05-6.72) more likely to implement the recommendation than the references. This finding is in line with a report of a study done in Addis Ababa, which revealed that respondents in the older age group were 6 times more likely to be adherent to recommendations than the younger age group (AOR = 5.72, 95% CI: 1.16, 28.14) [28]. The possible explanation could be due to older patients are believed to be more concerned about their health status and abide by caregiver’s advice to restrict themselves from risk factors that may affect their well-being. In contrast, this finding disagrees with reports from studies done in south Ethiopia [29], southwest Ethiopia [18], and Nigeria [17], which reported the older-aged group was less likely to practice recommendations compared to the younger group. This discrepancy might be due to differences in participants’ peer pressure, educational level, presence of comorbidities, and physical readiness among studies.

In this study, participants who attended primary school had higher odds of implementing recommendations than those who could not read and write (AOR: 3.84; 95% CI: 1.42-10.35). Participants who achieved college and above were 2.38 times (AOR: 2.38; 95% CI: 1.06-5.36) more likely to practice non-pharmacological recommendations than the counterparts. This finding is in agreement with the results of studies done in southwest Ethiopia [18], northeast Ethiopia [30], and Saudi Arabia [31], which identified that respondents who attended formal education were more likely to practice recommendations than respondents with no formal education. A possible justification is that an educated individuals can access information from various sources and believed to be more aware of the advantage of implementing non-pharmacological recommendations along with pharmacological therapy for hypertension control than the counter parts.

Participants with a monthly income exceeding five thousand Ethiopian Birr had lower odds of adhering to the non-pharmacological recommendations than their counterparts (AOR: 0.57; 95% CI: 0.34-0.96). This could be probably due to higher-income individuals in the setting might have sedentary occupations, nutrition transition with more access to processed foods, and less time for regular physical exercise. The finding is inconsistent with the reports of studies done in southern Ethiopia [29], northeastern Ethiopia [30], and Saudi Arabia [31]. This disagreement might be due to variation in participants’ job nature, social and cultural norms on food preferences, physical condition and availability of conducive setups and time to perform physical exercise among studies.

The current study identified that the study participants with good knowledge about non-pharmacological recommendations were five times (AOR: 5.02; 95% CI: 2.94-8.57) more likely to implement the recommendations than their counterparts. The finding is in agreement with the report of studies conducted in Addis Ababa [28], southern Ethiopia [29], southwest Ethiopia [18], and northeast Ethiopia [30]. This might be due to those with good knowledge about the recommendations having a better understanding of the advantage of each component of non-pharmacological recommendations for effective management of hypertension than their counterparts.

To our knowledge, no previous study has investigated the implementation of non-pharmacological strategies among hypertensive patients in the South Omo and Gofa zones. Therefore, this study findings would fill an existing information gap in the setting and would provide evidence for stakeholders and policy makers working toward hypertension management and prevention of its complications in hypertensive patients. However, this study has some limitations, such as recall bias due to use of self-report data, social desirability bias in which patients may over report positive behaviors and since cross-sectional design used, the study could not establish casual relationships.

## Conclusion and recommendations

This study revealed that about four in ten hypertensive patients poorly implemented non-pharmacological management recommendations. The study established that the likelihood of successful implementation is significantly predicted by the patient’s age, level of education, monthly income, and understanding of the non-pharmacological advice.

Therefore, we recommend establishing a facility-based patient peer support clubs to foster shared experiences and collective implementation; considering digital health intervention such as automated SMS reminders for literate patients and those with mobile phone access; organizing a community sensitization campaigns; and empowering health extension workers to provide patient targeted heath education to improve patients’ implementation of the non-pharmacological recommendation for hypertension management. Furthermore, future research needs to be conducted to investigate patients’ actual implementation of the non-pharmacological strategies through longitudinal study design to better capture patient’s real-world behaviors.

## Supporting information

S1 File The dataset used for this study. (SAV)
