# Supplementary material for: Implementation of non-pharmacological recommendations and its associated factors among hypertensive patients on follow-up in hospitals of South Omo and Gofa zones, Southern Ethiopia: A facility based cross sectional study
Source: PLOS Glob Public Health. 2026 Jun 10;6(6):e0006604. doi: 10.1371/journal.pgph.0006604 (PMC13252757; doi:10.1371/journal.pgph.0006604)
Supplement: Attachment — Submitted filename: Response to Reviewers.pdf [file pgph.0006604.s002.pdf]

Response to Reviewers

Dear esteemed Editor and Reviewers,

On behalf of all the authors and myself, I would like to extend my heartfelt appreciation for your indispensable scientific feedback you gifted us on our submission. The comments, suggestions and questions forwarded for clarification showcase how thoroughly you reviewed our manuscript line by line with due sacrification of your precious time. Dear all, we the authors positively accepted and incorporated the concerns forwarded and revised the manuscript accordingly with our utmost effort. We truly believe that your insightful feedbacks made our paper well-articulated, more readable and scientifically sound for the scientific community. The revisions made in the manuscript were highlighted by track changes and can be noted clearly in the revised submission. We have tried to reflect on some of the burning issues requiring clarification and consensus regarding the submission. Therefore, I kindly invite you to enjoy our reflections presented here below. Dear editor and reviewers, I want to make sure that we the authors are more open and ready to incorporate any further feedback you may forward on our revised submission.

Thank you in advance for your time and understanding!

Regarding the title revision:

Dear Editor,

We appreciate the comment given by Reviewer #1 on the manuscript title and the title revision request from the journal. Dear, for the sake of common understanding and consensus, we have kindly forwarded justification for our reservation or fear to revise the title as suggested and maintain the original title. The primary objective of this study was to assess the actual behavior (implementation/practice) of patients regarding non-pharmacological recommendations (e.g., salt reduction, exercise, weight management). In addition, it aims to identify factors associated with hypertensive patients' implementation of non-pharmacological recommendations. In this study, implementation of non-pharmacological recommendations is a dependent or outcome variable, and patients' knowledge and attitude towards the recommendations are included and assessed as part of independent variables along with patients' sociodemographic characteristics, medical history, and information access/source.

In this sense, we have conducted binary logistic regression analysis to check the presence or absence of a statistically significant association between the patients' implementation of non-pharmacological recommendations and independent variables such as patients' knowledge and attitude towards the recommendations and other factors. In this study, patients' knowledge level on recommendations has shown a significant association with the outcome variable, but attitude has not shown a significant association with the implementation of non-pharmacological recommendations in the final multivariable logistic regression analysis.

The proposed title focuses on Attitude and Awareness, which only measures what patients think or know. So, if we change the title as suggested, this will affect the components of the manuscript, including the methodology section, and may cause us to miss our primary objective.

The methodology employed was utilized to capture the magnitude of recommendation implementation among the hypertensive patients. The data collection tools and analysis were specifically designed to measure implementation levels of non-pharmacological recommendations. Revising the title to "Attitude and awareness would be technically inaccurate and would not accurately reflect the study. Because the "associated factors" identified in the study were analyzed in relation to implementation of non-pharmacological recommendations, not just to Attitude and Awareness.

To ensure the title remains a precise reflection of the data collected and the conclusions drawn, we the authors, maintain that "Implementation of non-pharmacological recommendations and its associated factors among hypertensive patients on follow-up in hospitals of South Omo and Gofa zones, Southern Ethiopia: A facility-based cross-sectional study'' is the most accurate and scientifically sound description of the work.

Therefore, we the authors, kindly request you to understand our reason why the manuscript is entitled ''Implementation of non-pharmacological recommendations and its associated factors among hypertensive patients on follow-up in hospitals of South Omo and Gofa zones, Southern Ethiopia: A facility-based cross-sectional study'' and let us proceed with the submission with the original title. Thank you for your understanding!

Financial Disclosure statement: We have not received grant to this work from any funder. The author(s) received no specific funding for this work.

Competing interests statement: update as per the suggestion. "The authors have declared that no competing interests exist."

Regarding data availability: We confirm that all the data and datasets used in this study are made available within the paper and in the supporting information.

Regarding Figure files: Figures were carefully revised as per the suggestions and format in the submission guideline.

Reviewer#1:

Regarding to the title modification:

Dear reviewer, we appreciate the comment you forwarded on the title and for the sake of common understanding and consensus, we have kindly forwarded justification for our reservation or fear to revise it as suggested and maintain the original title. The primary objective of this study was to assess the actual behavior (implementation/practice) of patients regarding non-pharmacological recommendations (e.g., salt reduction, exercise, weight management). In addition it is aimed to identify factors associated with hypertensive patients' implementation of non-pharmacological recommendations. In this study, implementation of non-pharmacological recommendations is a dependent or outcome variable and patients' knowledge and attitude towards the recommendations are included and assessed as part of an independent variables along with patients sociodemographic characteristics, medical history and information access/source.

In this sense, we have conducted binary logistic regression analysis to check presence or absence of statistically significant association between the patients' implementation of non-pharmacological recommendations and independent variables such as patients' knowledge and attitude towards the recommendations and other factors. In this study patients' knowledge level on recommendations have shown significant association with the outcome variable but attitude have not shown significant association with the implementation of non-pharmacological recommendations in final multivariable logistic regression analysis.

The proposed title focuses on Attitude and Awareness, which only measures what patients think or know. So, if we change the title as suggested, this will affect the entire components of the manuscript including the methodology section and may let us to miss our primary aim.

The methodology employed was utilized to capture the magnitude of recommendation implementation among the hypertensive patients. The data collection tools and analysis were specifically designed to measure implementation levels to non-pharmacological recommendations. Revising the title to “Attitude and awareness would be technically inaccurate and would not reflect the study. Because the "associated factors" identified in the study were analyzed in relation to implementation of non-pharmacological recommendations, not just to Attitude and Awareness.

To ensure the title remains a precise reflection of the data collected and the conclusions drawn, we the authors maintain that “Implementation of non-pharmacological recommendations and its associated factors among hypertensive patients on follow-up in hospitals of South Omo and Gofa zones, Southern Ethiopia: A facility-based cross-sectional study’’ is the most accurate and scientifically sound description of the work.

Therefore we the authors, kindly request you to understand our reason why the manuscript is entitled ‘’Implementation of non-pharmacological recommendations and its associated factors among hypertensive patients on follow-up in hospitals of South Omo and Gofa zones, Southern Ethiopia: A facility-based cross-sectional study’’ from its primary aim and allow as to maintain the submission with the original title.

Reference about the global burden of hypertensions: We updated it with the most recent (2024) data published in 2025 by WHO as suggested.

For references on guidelines for non-pharmacological treatment: We have revised and cited it with the relevant guideline for hypertension management in the revision.

Regarding sentence in line 88-89: To our knowledge, there is no previous study reporting hypertensive patients’ implementation of non-pharmacological recommendations in the current study area. Therefore, the sentence in line 88-89 is to highlight absence of the data particularly in the study area (South Omo and Gofa zones) not in the entire or other parts of the country.

Concern on variables: Sure, we have not mentioned blood pressure control and adherence to pharmacological therapy. Ideally these variables should be included but due to our focus on non-pharmacological therapy implementation we missed them and we take this as a draw back.

Data collection: Dear reviewer, our main intention was to assess patient’s implementation of non-pharmacological recommendation not to assess the blood pressure control though it is important.

Due to this, we have not focused on measuring and reporting blood pressure during data collection. Weight and height of the participants were measure to calculate BMI to check their weight management as one of the domains of the non-pharmacological strategy of hypertension therapy.

We incorporated the give comments given on the redundancy, table and use of phrases like nearly half and revised the paper accordingly.

Reviewer #2:

Regarding concerns on abstract section:

Introduction: revised according to the comment

Methods: Sampling technique included as per the suggestion.

Regarding the guideline: Dear reviewer, yes using the former over the latest could be taken as a drawback. But, the justification why we used it is that, in JNC7 guideline the non-pharmacological domains for hypertension management are clearly mentioned with due emphasis when we cross check it with the latest version. The JNC8 is the revised version and mainly focused on thresholds for defining hypertension and the selection of antihypertensive drug classes. JNC8 simply indicated that non-pharmacological strategies as an important component of therapy without mentioning the domains with updates in the guideline. Due to this, we used the former guideline during conception of our study and as per our knowledge concerns it has no effect on the study aim. But if any further concern on this, we are open to consider and update it accordingly.

Results: Dear, the suggestions incorporated in the revision accordingly. For the sake of managing word number limit in the abstract section we have been forced to highlight only the major findings in this section. Monthly income association with outcome is well explained in the main result and discussion sections accompanied with the possible justifications, why higher income is associated with lower odds of implementation.

Introduction section: Revised according to the given comments and suggestions.

Regarding concerns on methodology:

Sample size: Dear reviewer, the total calculate sample size was 376 but during data collection only 365 responded and the response rate in this study was 97.1% as indicated in the result section.

The 11 participants were refused to take part and withdrawal during data collection and taken as non-respondents. The number mentioned in abstract section is to indicate the number of participants from who the data were collected in the study not the total sample size. Therefore, in our revision, we avoided mentioning the number of participants in the abstract section to avoid such a potential confusion regarding to the sample size.

Sampling: Revised as suggested.

Tool: Dear reviewer, as we tried to explain above the JNC8 is the revised version and mainly focused on thresholds for defining and the selection of antihypertensive drug classes. JNC8 simply indicated that non-pharmacological strategies as an important component of therapy without mentioning the domains with updates in the guideline. Whereas in JNC7, all the non-pharmacological domains/components are clearly mentioned. Due to this reason, we used it over the revised one. If any further concern, we are ready to entertain in the future revision.

Regarding operational definition for implementation: Dear reviewer, we accept the given comment and it can be taken as a methodological weakness as you said. But for common understanding, the other related studies used mean value to define the status of patients practice or implementation by calculating mean score for participant’s response to practice or implementation assessing questions. To discuss our find with the others related studies we defined the implementation status in the same manner in our study just to make it consistent with the previous works. Otherwise if defined in different way it would be difficult to discuss it with the related studies.

Regarding comments on result section: We revised this section according to the suggestions. Table 5 was reconstructed to make it clear and avoid confusion. Why we did not included AOR with P-values for factors in the text is just to avoid redundancy because they can be seen easily from the Table 6 and narrated in the discussion section while comparing with others work.

Fig. 2" is mentioned but not provided in this section: based on the journals submission guideline figures are submitted separately this why figure are not available immediately at the location of citation and made available after the reference section in the submitted PDF of the manuscript.

Comment of discussion section: Dear reviewer, we have revise it as per the give indispensable suggestions.

Conclusion and recommendations: Dear reviewer, we incorporated the suggestions and revise it according to the given comments.

Reviewer #3:

1: Introduction section: Dear reviewer, we revised it carefully as per the suggestion.

2. Table 5: Dear, Table 5 is corrected and restructured in the revision.

3. Discussion: Dear, we incorporated the suggestions in the revision.

Thank you so much for time and scientific inputs!
