# Supplementary material for: Implementation of non-pharmacological recommendations and its associated factors among hypertensive patients on follow-up in hospitals of South Omo and Gofa zones, Southern Ethiopia: A facility based cross sectional study
Source: PLOS Glob Public Health. 2026 Jun 10;6(6):e0006604. doi: 10.1371/journal.pgph.0006604 (PMC13252757; doi:10.1371/journal.pgph.0006604)
Supplement: Peer review history

# Peer review: Decision Letter 0

**DOI:** 10.1371/journal.pgph.0006604.r001

**Authors:** Harry H.X. Wang

3 Mar 2026

PGPH-D-25-03900

Implementation of non-pharmacological recommendations and its associated factors among hypertensive patients on follow-up in hospitals of South Omo and Gofa zones, Southern Ethiopia: A facility-based cross-sectional study

PLOS Global Public Health

Dear Dr. Kanko,

Thank you for submitting your manuscript to PLOS Global Public Health. After careful consideration, we feel that it has merit but does not fully meet PLOS Global Public Health’s publication criteria as it currently stands. Therefore, we invite you to submit a revised version of the manuscript that addresses the points raised during the review process.

Please submit your revised manuscript by Apr 17 2026 11:59PM. If you will need more time than this to complete your revisions, please reply to this message or contact the journal office at globalpubhealth@plos.org. When you're ready to submit your revision, log on to https://www.editorialmanager.com/pgph/ and select the 'Submissions Needing Revision' folder to locate your manuscript file.

Please include the following items when submitting your revised manuscript:

- A letter that responds to each point raised by the editor and reviewer(s). You should upload this letter as a separate file labeled 'Response to Reviewers'.
- A marked-up copy of your manuscript that highlights changes made to the original version. You should upload this as a separate file labeled 'Revised Manuscript with Track Changes'.
- An unmarked version of your revised paper without tracked changes. You should upload this as a separate file labeled 'Manuscript'.

Guidelines for resubmitting your figure files are available below the reviewer comments at the end of this letter.

We look forward to receiving your revised manuscript.

Kind regards,

Harry H.X. Wang, PhD

Guest Editor

PLOS Global Public Health

Journal Requirements:

1. During your revisions, please revise your title in line with the reviewer request. we liek to suggest: "Attitude and awareness on non-pharmacological treatment and its associated factors among hypertensive patients on follow-up in hospitals of South Omo and Gofa zones, Southern Ethiopia: A facility-based cross-sectional study." Please ensure that the title is updated it in the manuscript file and online submission information if needed, and is an accurate reflection of the study."

2. Please provide a detailed online Financial Disclosure statement. This is published with the article. It must therefore be completed in full sentences and contain the exact wording you wish to be published.

a) State the initials, alongside each funding source, of each author to receive each grant. For example: “This work was supported by the National Institutes of Health (####### to AM; ###### to CJ) and the National Science Foundation (###### to AM).”

b) State what role the funders took in the study. If the funders had no role in your study, please state: “The funders had no role in study design, data collection and analysis, decision to publish, or preparation of the manuscript.”

For more information, please go to our submission guidelines:

https://journals.plos.org/globalpublichealth/s/submission-guidelines#loc-financial-disclosure-statement

3. Please ensure that the funders and grant numbers match between the Financial Disclosure field and the Funding Information tab in your submission form. Note that the funders must be provided in the same order in both places as well.

4. Please update your online Competing Interests statement. If you have no competing interests to declare, please state: "The authors have declared that no competing interests exist."

5. We note that your Data Availability Statement is currently as follows: "All relevant data are available within the paper and supporting information."

Please confirm at this time whether or not your submission contains all raw data required to replicate the results of your study. Authors must share the “minimal data set” for their submission. PLOS defines the minimal data set to consist of the data required to replicate all study findings reported in the article, as well as related metadata and methods (https://journals.plos.org/globalpublichealth/s/data-availability#loc-minimal-data-set-definition).

For example, authors should submit the following data:

- The values behind the means, standard deviations and other measures reported;

- The values used to build graphs;

- The points extracted from images for analysis.

Authors do not need to submit their entire data set if only a portion of the data was used in the reported study.

If your submission does not contain these data, please either upload them as Supporting Information files or deposit them to a stable, public repository and provide us with the relevant URLs, DOIs, or accession numbers. For a list of recommended repositories, please see https://journals.plos.org/globalpublichealth/s/recommended-repositories.

If there are ethical or legal restrictions on sharing a de-identified data set, please explain them in detail (e.g., data contain potentially sensitive information, data are owned by a third-party organization, etc.) and who has imposed them (e.g., an ethics committee). Please also provide contact information for a data access committee, ethics committee, or other institutional body to which data requests may be sent. If data are owned by a third party, please indicate how others may request data access.

6. Please ensure that the Title in your manuscript and the Title in your online submission form are the same.

7. Please provide separate main figure files in .tif or .eps format only and ensure that all files are under our size limit of 10MB.

For more information about how to convert your figure files please see our guidelines: https://journals.plos.org/globalpublichealth/s/figures

8. Please include a separate legend or caption for each figure in your main manuscript.

9. If the reviewer comments include a recommendation to cite specific previously published works, please review and evaluate these publications to determine whether they are relevant and should be cited. There is no requirement to cite these works unless the editor has indicated otherwise.

Additional Editor Comments (if provided):

[Note: HTML markup is below. Please do not edit.]

Reviewers' comments:

Reviewer's Responses to Questions

**Comments to the Author**

1. Does this manuscript meet PLOS Global Public Health’s publication criteria? Is the manuscript technically sound, and do the data support the conclusions? The manuscript must describe methodologically and ethically rigorous research with conclusions that are appropriately drawn based on the data presented.

Reviewer #1: Partly

Reviewer #2: Yes

Reviewer #3: Partly

**********

2. Has the statistical analysis been performed appropriately and rigorously?

Reviewer #1: Yes

Reviewer #2: Yes

Reviewer #3: Yes

**********

3. Have the authors made all data underlying the findings in their manuscript fully available (please refer to the Data Availability Statement at the start of the manuscript PDF file)?

The PLOS Data policy requires authors to make all data underlying the findings described in their manuscript fully available without restriction, with rare exception. The data should be provided as part of the manuscript or its supporting information, or deposited to a public repository. For example, in addition to summary statistics, the data points behind means, medians and variance measures should be available. If there are restrictions on publicly sharing data—e.g. participant privacy or use of data from a third party—those must be specified.

Reviewer #1: Yes

Reviewer #2: Yes

Reviewer #3: Yes

**********

4. Is the manuscript presented in an intelligible fashion and written in standard English?

PLOS Global Public Health does not copyedit accepted manuscripts, so the language in submitted articles must be clear, correct, and unambiguous. Any typographical or grammatical errors should be corrected at revision, so please note any specific errors here.

Reviewer #1: Yes

Reviewer #2: Yes

Reviewer #3: No

**********

5. Review Comments to the Author

Please use the space provided to explain your answers to the questions above. You may also include additional comments for the author, including concerns about dual publication, research ethics, or publication ethics. (Please upload your review as an attachment if it exceeds 20,000 characters)

Reviewer #1: Overall, this is a report of hypertensive patients' attitude and awareness on non-pharmacological treatment, rather than the objective proof of implementation of those recommendations.

I would suggest modifying the title accordingly.

The general references about the global prevalence of hypertensions are outdated. It is a suggestion to cite newer data from the Global Burden of Disease studies and NCD group (NCD Risk Factor Collaboration (NCD-RisC). Worldwide trends in hypertension prevalence and progress in treatment and control from 1990 to 2019: a pooled analysis of 1201 population-representative studies with 104 million participants. Lancet. 2021 Sep 11;398(10304):957-980.

For references on guidelines for non-pharmacological treatment, please cite relevant international guidelines (by AHA, ISH, European cardiology guidelines for prevention etc).

In section Introduction, line 88,89 the statement that there are no data reporting on the level of implementation of lifestyle strategies may not be correct. Please look and preferably consider including Kebede T, Taddese Z, Girma A. Knowledge, attitude and practices of lifestyle modification and associated factors among hypertensive patients on-treatment follow up at Yekatit 12 General Hospital in the largest city of East Africa: A prospective cross-sectional study. PLoS One. 2022 Jan 27;17(1):e0262780.

In study variables, among reported, there is no mention of blood pressure control levels. Adherence to pharmacological therapy was not assessed, and it is feasible work-up in hospital outpatient settings.

In Data collection tool and procedure beside self-reported quiesstionaire the subject hight and weight was measured, but not office blood pressure. Please explain the reason for omitting this crucial procedure when reporting on hypertensive patients.

Please avoid frequent use of ‘’nearly half, nearly third, almost half, etc. This is imprecise, misleading and inappropriate when giving exact percentages in brackets. The rough numbers in text with percentages are redundant and are shown in tables.

Consider that paradoxically higher income (but not higher educational level) may be due to slectional bias, rather than psychological perculiarities of hypertensive patients in this area (egg. referrals to hospital center, severeness of hypertension, the number of comorbidities).

Instead of numerous confusing tables, consider reporting data in other graphical formats for clarity.

Reviewer #2: Reviewer Report

Manuscript ID: PGPH-D-25-03900

Title: Implementation of non-pharmacological recommendations and its associated factors among hypertensive patients on follow-up in hospitals of South Omo and Gofa zones, Southern Ethiopia: A facility based cross sectional study

Overall Comment

This manuscript addresses an important and under-researched topic with a well-structured methodology, clear objectives, and robust statistical analysis. The study provides valuable baseline data for the region and is generally well-written. However, it suffers from several critical issues: inconsistencies in sample size reporting, an overly generic conclusion that does not fully address all significant predictors, and a discussion section that relies on repetitive, superficial justifications for findings. The arbitrary use of mean scores to define "good" implementation and the omission of key limitations (social desirability bias, cross-sectional design) further weaken the scientific rigor.

Specific comments

1. Abstract

• Overall the structure is good

Background:

• The background is slightly wordy and could be more direct.

Methods:

• Does not mention the sampling technique (e.g., systematic random sampling?).

• Using JNC7 (dated 2003) as a primary reference is a limitation, as guidelines have evolved.

Results:

• Include sociodemographic factors at least gender and age

• Does not state the p-values (just says <0.05).

• The AOR for income (0.57) suggests it is a protective factor, but the narrative doesn't explain this direction.

Conclusion and Recommendation

• The recommendation is too generic. It focuses solely on "health education," even though factors like age and monthly income were also significant. A more multifaceted recommendation would be stronger.

2. Introduction

Overall, the introduction follows a standard flow structure (global → local) and establishes the topic well.

Area of improvement

• It is too lengthy, repetitive, and lacks a sharp, evidence-based problem statement for the specific study area.

• Lines 66-79 repeat definitions of non-pharmacologic management multiple times; this could be condensed significantly.

• Line 88 states "no data" for the study setting, but it does not explain why the South Omo and Gofa zones might have unique factors (e.g., cultural practices, healthcare access) that justify the study. The gap is stated, but not contextualized.

• The final sentence (lines 89-93) is a single, overly long run-on sentence. It lists importance but does not explicitly state the aims of the study

• Line 52-53: "Now a days it is a global challenge..." – awkward phrasing; "Currently" would be better.

• Does not discuss the consequences of poor implementation specifically in the Ethiopian context (e.g., impact on the already strained healthcare system).

3. Method

• Overall, the methodology is generally sound and well-structured, but contains several critical errors and inconsistencies, that must be addressed before publication.

Area of improvement

• Lines 111-112 vs. Abstract: The final sample size here is 376 (after adjusting for non-response). However, the Abstract (Line 25) states the study was conducted among 365 patients. There is a discrepancy of 11 participants. The manuscript must explain what happened to these 11 (were they the non-response?).

• The section never explicitly states the actual final number of participants analyzed (appears to be 365 based on abstract, but not stated here).

• Line 116-119 (Sampling): The "lottery method" is mentioned, but the text does not explicitly state that simple random sampling was used. It should be clearly named.

• Line 134-140 (Tool): Citing JNC7 (2003) is a limitation. While acceptable for historical comparison, justifying why an older guideline was used over more recent ones (JNC8) would strengthen the text.

• Line 177-182 (Operational Definition – Implementation): Using the mean (5.8) as a cut-off for "good" vs "poor" is arbitrary. "Good" implementation should ideally be defined by clinical guidelines (e.g., meeting all recommendations), not statistical averages. This is a methodological weakness.

4. Result

• Overall, the results are comprehensive and well-organized, with clear tables and logical flow. However, several tables contain data that are merely repeated in text without added value.

Area for improvement

• Redundancy (Lines 237-242, 247-252): The text simply repeats what is already visible in the tables. For example, lines 239-242 list percentages that are already in Table 3. This adds no value. Solution: Summarize patterns instead of repeating numbers (e.g., "Most participants recognized the risks of high salt intake and smoking, but knowledge about specific dietary fats was lower.").

• Line 270 mentions "information access" and "family support" were significant in bivariable analysis, but these are not clearly defined in the variables section.

• Some tables lack clarity for instance Table 5: The table is confusing. It mixes frequency data (How often...) with yes/no data (Do you perform...) in a way that is hard to follow. The layout could be restructured.

• Income Finding (Line 275): The AOR for income (0.57, 0.49, 0.44) shows that higher income is associated with lower odds of good implementation. This is counterintuitive and deserves mention in the text, but it is simply listed without comment.

• P-values missing in Text: While the table shows p-values, the text (Lines 273-276) does not restate them. For readability, key p-values should be mentioned alongside the AOR.

• Line 256: "Fig. 2" is mentioned but not provided in this section.

5. Discussion

• Overall, the discussion is well-structured, compares findings with appropriate literature, and addresses the study objectives. However, it is too descriptive, contains weak justifications, and overstates the "first of its kind" claim without acknowledging several critical limitations.

Area need improvement

• Weak Justifications for Variations (Lines 296-300, 312-313, 331-332): The repeated explanation—"differences in educational level, economic status, awareness, and cultural factors"—is generic and overused. It appears for almost every discrepancy without specific insight into why these particular contexts differ. This weakens the analytical depth.

• Not clear Income Interpretation (Lines 325-332): The finding that higher income is associated with lower implementation is dismissed as a "paradox" with a weak speculation ("lack of knowledge or unwillingness"). This deserves deeper exploration. Possible explanations (e.g., higher-income individuals have sedentary jobs, more access to processed foods, less time for exercise) are not considered.

• The discussion mentions only recall bias (Line 343-344). Several major limitations are ignored: Cross-sectional design: Cannot establish causality; Social desirability bias: Patients may over report good behaviors; Use of JNC7 guidelines: Outdated at the time of writing;

• Overstatement (Line 341): "This research is the first of its kind in the study area" is a strong claim. While likely true for these specific zones, it sounds self-promotional. A softer phrasing ("To our knowledge, no previous study has examined this in South Omo and Gofa zones") is more appropriate.

• The discussion does not translate findings into practical recommendations for clinicians or policymakers in the study setting.

6. Conclusion and Recommendations

• In general the conclusion accurately summarizes the main findings, but the recommendations are too narrow, generic, and do not logically flow from the full set of significant predictors. The section misses an opportunity to provide targeted, actionable advice.

Area of improvement

• Recommendation-Predictor Mismatch (Lines 350-353): The recommendation focuses only on health education (addressing knowledge). However, the study found that age and monthly income were also significant predictors. Health education alone will not help older patients or those with lower income if structural barriers exist. This is a logical disconnect.

• Lines 350-351: "Health education... using various approaches, such as digital alternatives" is too vague. What specific approaches? For whom? Digital alternatives may not reach older, low-income, or rural populations (who were significant in this study).

• Given the findings, recommendations should have targeted such as Individual level, Health system level, so on

• Suggest differentiated health education (e.g., peer support for older adults, digital tools for younger, employed patients).

• Recommend further research into why higher income was associated with lower implementation.

Reviewer #3: Dear editor, thank you for the invitation as a reviewer.

Here is my comment:

• Overall, the paper is good, addressing one of the NCDs in our country

1. In the introduction section,

• add a strong rationale for why it is important to study or work on non-pharmacological therapy

2. Table 5: Study participants’ implementation of non-pharmacological recommendations for hypertension management (n=365)

• The presentation of variables has to be uniform and sequential. It is confusing

3. Discussion

• The rationale for the factors associated needs further analysis/ elaboration, e.g., income increment… it seems that sociocultural factors, practical aspects have to be analyzed

• Other factors also need further contextual elaborations

**********

6. PLOS authors have the option to publish the peer review history of their article (what does this mean?). If published, this will include your full peer review and any attached files.

**Do you want your identity to be public for this peer review?** If you choose “no”, your identity will remain anonymous but your review may still be made public.

For information about this choice, including consent withdrawal, please see our Privacy Policy.

Reviewer #1: No

Reviewer #2: No

Reviewer #3: No

**********

[NOTE: If reviewer comments were submitted as an attachment file, they will be attached to this email and accessible via the submission site. Please log into your account, locate the manuscript record, and check for the action link "View Attachments". If this link does not appear, there are no attachment files.]

-->Figure Resubmissions:

While revising your submission, we strongly recommend that you use PLOS’s NAAS tool (https://ngplosjournals.pagemajik.ai/artanalysis) to test your figure files. NAAS can convert your figure files to the TIFF file type and meet basic requirements (such as print size, resolution), or provide you with a report on issues that do not meet our requirements and that NAAS cannot fix.

After uploading your figures to PLOS’s NAAS tool - https://ngplosjournals.pagemajik.ai/artanalysis, NAAS will process the files provided and display the results in the "Uploaded Files" section of the page as the processing is complete. If the uploaded figures meet our requirements (or NAAS is able to fix the files to meet our requirements), the figure will be marked as "fixed" above. If NAAS is unable to fix the files, a red "failed" label will appear above. When NAAS has confirmed that the figure files meet our requirements, please download the file via the download option, and include these NAAS processed figure files when submitting your revised manuscript.

# Author comment: Author response to Decision Letter 1

**DOI:** 10.1371/journal.pgph.0006604.r002

9 Apr 2026

Attachment Submitted filename: Response to Reviewers.pdf

# Peer review: Decision Letter 1

**DOI:** 10.1371/journal.pgph.0006604.r003

**Authors:** Harry H.X. Wang

20 May 2026

Implementation of non-pharmacological recommendations and its associated factors among hypertensive patients on follow-up in hospitals of South Omo and Gofa zones, Southern Ethiopia: A facility-based cross-sectional study

PGPH-D-25-03900R1

Dear Mr Kanko,

We are pleased to inform you that your manuscript 'Implementation of non-pharmacological recommendations and its associated factors among hypertensive patients on follow-up in hospitals of South Omo and Gofa zones, Southern Ethiopia: A facility-based cross-sectional study' has been provisionally accepted for publication in PLOS Global Public Health.

Before your manuscript can be formally accepted you will need to complete some formatting changes, which you will receive in a follow up email. A member of our team will be in touch with a set of requests.

Please note that your manuscript will not be scheduled for publication until you have made the required changes, so a swift response is appreciated.

IMPORTANT: The editorial review process is now complete. PLOS will only permit corrections to spelling, formatting or significant scientific errors from this point onwards. Requests for major changes, or any which affect the scientific understanding of your work, will cause delays to the publication date of your manuscript.

If your institution or institutions have a press office, please notify them about your upcoming paper to help maximize its impact. If they'll be preparing press materials, please inform our press team as soon as possible -- no later than 48 hours after receiving the formal acceptance. Your manuscript will remain under strict press embargo until 2 pm Eastern Time on the date of publication. For more information, please contact globalpubhealth@plos.org.

Thank you again for supporting Open Access publishing; we are looking forward to publishing your work in PLOS Global Public Health.

Best regards,

Harry H.X. Wang PhD, FAcadTM, ISHF, IFJSH

Professor, Sun Yat-Sen University

Guest Editor, PLOS Global Public Health

***********************************************************

Reviewer Comments (if any, and for reference):

Reviewer's Responses to Questions

**Comments to the Author**

1. If the authors have adequately addressed your comments raised in a previous round of review and you feel that this manuscript is now acceptable for publication, you may indicate that here to bypass the “Comments to the Author” section, enter your conflict of interest statement in the “Confidential to Editor” section, and submit your "Accept" recommendation.

Reviewer #2: All comments have been addressed

**********

2. Does this manuscript meet PLOS Global Public Health’s publication criteria? Is the manuscript technically sound, and do the data support the conclusions? The manuscript must describe methodologically and ethically rigorous research with conclusions that are appropriately drawn based on the data presented.

Reviewer #2: Yes

**********

3. Has the statistical analysis been performed appropriately and rigorously?

Reviewer #2: Yes

**********

4. Have the authors made all data underlying the findings in their manuscript fully available (please refer to the Data Availability Statement at the start of the manuscript PDF file)?

The PLOS Data policy requires authors to make all data underlying the findings described in their manuscript fully available without restriction, with rare exception. The data should be provided as part of the manuscript or its supporting information, or deposited to a public repository. For example, in addition to summary statistics, the data points behind means, medians and variance measures should be available. If there are restrictions on publicly sharing data—e.g. participant privacy or use of data from a third party—those must be specified.

Reviewer #2: Yes

**********

5. Is the manuscript presented in an intelligible fashion and written in standard English?

PLOS Global Public Health does not copyedit accepted manuscripts, so the language in submitted articles must be clear, correct, and unambiguous. Any typographical or grammatical errors should be corrected at revision, so please note any specific errors here.

Reviewer #2: Yes

**********

6. Review Comments to the Author

Please use the space provided to explain your answers to the questions above. You may also include additional comments for the author, including concerns about dual publication, research ethics, or publication ethics. (Please upload your review as an attachment if it exceeds 20,000 characters)

Reviewer #2: Manuscript ID: PGPH-D-25-03900

Title: Implementation of non-pharmacological recommendations and its associated factors among hypertensive patients on follow-up in hospitals of South Omo and Gofa zones, Southern Ethiopia: A facility based cross sectional study

Thank you for the opportunity to review the revised version of this manuscript.

I have carefully examined the authors’ responses and the revised manuscript. I am pleased to note that the authors have adequately addressed all the comments and concerns raised during the initial review. The revisions have substantially improved the clarity, methodological consistency, discussion depth, and overall scientific rigor of the manuscript.

In particular, the authors have appropriately clarified the methodological inconsistencies, strengthened the interpretation of key findings, improved the discussion and conclusion sections, and incorporated the suggested limitations and targeted recommendations.

At this stage, I have no additional comments or concerns. In my opinion, the manuscript is suitable for publication in its current revised form.

**********

7. PLOS authors have the option to publish the peer review history of their article (what does this mean?). If published, this will include your full peer review and any attached files.

**Do you want your identity to be public for this peer review?** If you choose “no”, your identity will remain anonymous but your review may still be made public.

For information about this choice, including consent withdrawal, please see our Privacy Policy.

Reviewer #2: No

**********
